# Acoustic Noise-Based Detection of Ferroresonance Events in Isolated Neutral Power Systems with Inductive Voltage Transformers

**DOI:** 10.3390/s23010195

**Published:** 2022-12-24

**Authors:** Raquel Martinez, Alberto Arroyo, Alberto Pigazo, Mario Manana, Eduardo Bayona, Francisco J. Azcondo, Sergio Bustamante, Alberto Laso

**Affiliations:** 1 Departamento de Ingeniería Eléctrica y Energética, Universidad de Cantabria, Av. Los Castros s/n, 39005 Santander, Spain; 2 Departamento de Ingeniería Informática y Electrónica, Universidad de Cantabria, Av. Los Castros s/n, 39005 Santander, Spain; 3 Departamento de Ingeniería Electromecánica, Tecnología Electrónica, Universidad de Burgos, Av. Cantabria s/n, 09006 Burgos, Spain; 4 Departamento de Tecnología Electrónica Ingeniería de Sistemas y Automática, Universidad de Cantabria, Av. Los Castros s/n, 39005 Santander, Spain

**Keywords:** ferroresonance detection, inductive voltage transformer, potential transformer

## Abstract

Power-quality events and operation transients in power systems (PS) with isolated neutral can saturate inductive voltage transformers (IVT), which, when interacting with the overhead and underground cable capacitances, can cause ferroresonance events in the local PS. This abnormal operating mode can partially or totally damage the transformers and switchgears within the affected PS. Distribution system operators (DSO) can minimize these effects by detecting ferroresonance events accurately and fast enough and changing the mode of operation accordingly. Direct detection methods, i.e., based on voltage measurements, are reliable, but the massive deployment of this solution is relatively expensive; i.e., power quality analyzers cost thousands of USD. Alternatively, indirect detection methods are also available, e.g., IVT vibration measurements with accelerometers costing hundreds of USD, but their reliability depends on the installation method used. This manuscript proposes using the acoustic noise caused by magnetostriction forces within the IVT core during ferroresonance events to detect their occurrence. Compared to other indirect methods, electret condenser microphones with preamplifying stage cost less than USD 10 and are less sensitive to the installation procedure. The proposed method is validated experimentally, and its performance compared to IVT vibration measurements one by using the same detection methodology.

## 1. Introduction

Ferroresonance events occur in isolated neutral power systems (PS) due to the series [1] or parallel configuration of capacitances, e.g., parasitic capacitances of the switches, overhead and underground power lines, and saturable inductances (mainly in both inductive voltage and power transformers) [2]. In addition, the system needs an energy source, low losses, and the appearance of a transient to trigger the ferroresonance. The type of ferroresonance depends on the nature of the transients and the power system characteristics [3]. Transients due to switching operations can result in series ferroresonance events due to stray capacitances of breakers, while transients created by faults can produce parallel ferroresonance due to the line-to-ground capacitances of the lines.

The effects of ferroresonances on inductive voltage transformers (IVTs) are well-known and include core saturation, current and voltage distortion [4], overcurrents and overvoltages [5], temperature raises, vibration, noise, damages in the primary winding [6], and the displacement of the neutral potential of the transformers outside the normal voltage triangle [7]. Recent studies also establish severe effects on wind turbines with doubly fed induction generators (DFIG) [8,9], which become catastrophic if superimposed on subsynchronous resonances (SSR) [10].

To prevent the appearance of ferroresonances, mitigation systems can be used on the IVT side. The most widespread approach is passive and consists of a burden resistor connected to the protection winding of the IVT. However, selecting the right resistor value depends on the PS characteristics [11], and large loads can damage the IVT due to excess dissipated power during long-term faults. More advanced passive solutions, consisting of controlled variable resistors [12] or saturable reactors [13], can be connected to dissipate the ferroresonance energy after its appearance, but, as a limitation, their ability to adjust the damping action is limited and they cannot be started prematurely to avoid the ferroresonance. Active damping systems try to overcome these problems [14].

On the grid side, limiting the line capacitance [15] contributes to preventing the occurrence of ferroresonances, and, after their appearance, isolating the area with ferroresonance and changing the grid topology within this area (e.g., disconnecting long lines and distributed generation systems) facilitate the mitigation of the event [16]. In the case of occurrence within wind farms [17], STATCOMs can be used to mitigate them and protect the wind turbines [18]. Furthermore, the malfunction of overcurrent relays due to ferroresonances can be prevented by coordinating identification and relay actions [19].

A sufficiently accurate and fast detection of the appearance of ferroresonances is crucial, on both the grid and the IVT sides, most of the detection methods available in the technical literature are based on the direct measurement of electrical variables, e.g., voltage waveforms. These detection methods can be integrated into active damping systems, as in [20], resulting in a fast damping time, typically hundreds of ms, or more sophisticated and dedicated measuring equipment can be used, e.g., power quality analyzers (PQA).

The IVT voltage waveforms are used in [21] to estimate the magnetic flux within the IVT core. Once filtered out, the subharmonic components are identified and added to be compared to a threshold level. If this value is exceeded for 100 ms, ferroreresonance is detected. Similarly, in [22], the voltage waveforms are processed with wavelet filters. By identifying the accumulation of energy at different levels and scales of decomposition, the threshold level for ferroresonance detection is set. In [23,24,25], the voltage waveforms, also decomposed through wavelets, are then processed by means of artificial neural networks (ANN). The results given show effectiveness higher than 93%, 95% and 99%. The approach in [26] is slightly different: Fourier analysis is used with voltage waveform samples, and the results for the fundamental and certain subharmonics are compared to previously defined values. In [27], the IVT voltages and currents are measured at the primary level to determine the saturation coefficient of the iron core of the IVT, and upon reaching a certain threshold, the ferroresonance is identified from line-to-ground faults. Iron core saturation is also used in [28,29] to reveal the appearance of a ferroresonance.

However, the deployment of the appropriate sensors and relays to implement the detection methods can be relatively expensive due to precision, sensitivity, and insulation requirements. Detection times can be less restrictive at the grid level since protection operations, e.g., changing the grid topology and de-energizing, require from hundreds of ms to s. This would potentially allow indirect detection methods to be used.

For indirect detection methods, experiments on temperature variations of IVTs due to ferroresonances have been carried out in [30]. The obtained results show that the IVT heating occurs in an interval of hours. In [31], it is shown that the IVT heating increases with the line capacitance, and, therefore, preventing the ferroresonance by reducing the length of the line would make its detection more difficult by measuring the IVT temperature. Alternatively, in [32], the IVT vibrations during ferroresonance events are sensed through a 0.3–18 kHz accelerometer, and the proposed detection algorithm is implemented in a 8-bit 16 MHz µC. The solution is cost-effective and the detection times are appropriate for grid operations during these events. Drawbacks include the fact that the performance of the method is sensitive to the installation of the accelerometer on the IVT case and that one accelerometer must be used per IVT. The changes in the IVT noise pattern are also associated with the phenomenon of ferroresonance, which is distinctively different and louder than the normal operation noise [33], due to the magnetostriction of the steel and the movements of the core laminae. Acoustic signals have been proposed in [34] for conductor-identification operations using an acoustic signal generator and receiver, reducing both the duration of these tests and the number of operations required.

This manuscript proposes overcoming the limitations for the deployment of the indirect detection method in [32] by replacing the accelerometers placed on the IVT case with in-switchgear microphones. The proposed method requires one sensor per switchgear, which is cost-effective and is less sensitive to human error during installation. The principles of the detection algorithm are those already published in [32], but the threshold levels must be adjusted to operate with the novel acoustic noise sensor. In Section 2, the mathematical foundations of the ferroresonance phenomena are given, together with the detection methodology proposed in [32]. Considerations on the use of acoustic noise to detect ferroresonance events are given in Section 3. The novel indirect detection method is validated experimentally by means of the laboratory setup, described in Section 4, and the results obtained with both accelerometers and low-cost microphones, with different locations, are provided in Section 5. Finally, a discussion is given.

## 2. Indirect Ferroresonances Detection in MV Isolated-Neutral Power Systems

### 2.1. Phenomenon Description

The occurrence of a ferroresonance event requires, at least, a saturable inductance, a capacitance, an energy source, and low system losses. The electric grid has many saturable inductances, underground and overhead lines, capacitances, power transformers, IVTs for measurement, shunt reactors, capacitive transformers, and capacitor banks. Ferroresonance is stimulated by both electrical resonances and transient events (i.e., overvoltages due to lightning), line faults, and switching operations.

Series ferroresonances are common when the phenomenon triggering it is due to switching operations in one or two of the phases, keeping the non linear inductance of the IVT in series with the capacitance of the switches. Parallel ferroresonances commonly occur in isolated neutral power systems when the triggering event is a line-to-ground fault. In this case, the non-linear inductances (IVTs, shunt reactances, etc.) are in parallel with the capacitances (power lines, capacitor banks, etc.). The analysis in this paper focuses on the detection of ferroresonance events in isolated neutral power systems; therefore, only parallel ferroresonance are analyzed.

**Figure 1:**
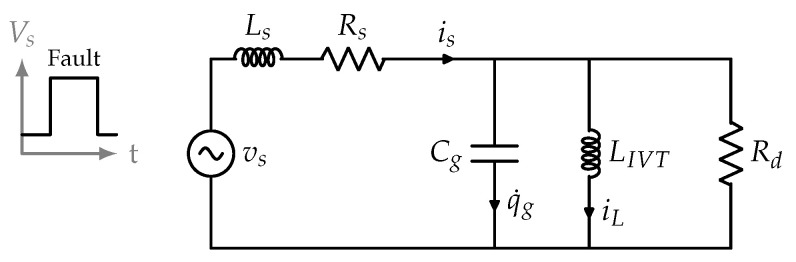
Per-phase equivalent circuit for parallel ferroresonance analysis.

The occurrence of ferroresonances in inductive voltage transformers for MV isolated neutral distribution power systems stimulated by line-to-ground faults can be analyzed by means of the equivalent per-phase circuit in Figure 1. At the primary IVT, the power system can be modeled by its Thevenin equivalent, where $L_{s}$ and $R_{s}$ are due to the power system characteristics and $v_{s}$ models the equivalent line-to-ground voltage, with RMS value $V_{s}$. The equivalent capacity to the ground at the IVT primary can be modeled through $C_{g}$, and its value also depends on the line and power system characteristics. The saturable magnetization inductance of the IVT is modeled through $L_{IVT}$ and can be obtained from the manufacturers’ data sheet or tests carried out by the DSO. Secondary loads of the IVT from the primary are modeled through $R_{d}$. It must be considered that most of $R_{d}$ is due to the damping resistor connected to the protection secondary.

From the equivalent circuit in Figure 1, and neglecting the effect of $R_{d}$, the following phasorial relationship is established:

(1) $${\bar{I}}_{s}\approx {\bar{I}}_{C_{g}}+{\bar{I}}_{L}=\frac{{\bar{V}}_{s}}{{\bar{X}}_{C_{g}}}+\frac{{\bar{V}}_{s}}{{\bar{X}}_{L_{IVT}}}$$

**Figure 2:**
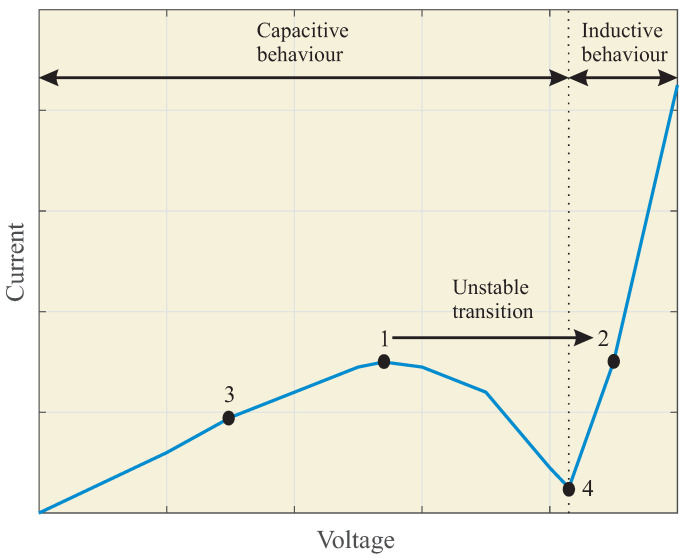
Non-linear behavior of the parallel ferroresonance event in isolated neutral power systems.

Figure 2 depicts the inductive and capacitive behaviors of the power system current, according to Equation (1), depending on the IVT primary voltage, ${\bar{V}}_{s}$. The operation point #3 corresponds to the normal operation condition. By increasing the IVT primary voltage, point #1 is reached, where the impedance is zero and negative beyond this point, which leads to a non-stable condition. Since the current levels increase due to line-to-ground faults, the IVT operation point jumps towards the operation point #2. At this new operation point, the ferroresonance event is maintained over time, if the power system remains energized. Point #4 in Figure 2 corresponds to the limit between capacitive and inductive behaviors.

The non-linearity of the equivalent circuit in Figure 1 can also be evaluated through a set of differential equations. Considering that the current through the saturable $L_{IVT}$ depends on the transformer magnetic flux ϕ(t), and is given by

(2) $$i_{L}\left(t\right)=a\phi \left(t\right)+b\phi^{p}\left(t\right),$$

with coefficients *a*, *b*, and *p*, allows the IVT behavior to be adjusted to the given characteristics. Modern inductive IVT used by DSOs at the 20 kV level can be usually adjusted with $a\in (10^{-5},10^{-4})$, $b\in (10^{-10},10^{-5})$ and p=5.

Due to the occurrence of a line-to-ground fault, the voltage triangle shifts and the IVT primary voltage increases, reaching the saturation region, as depicted in Figure 2.

From Figure 1, the following equations are verified across the whole transient

(3) $$\left\{\begin{array}{c} {\dot{i}}_{s}\left(t\right)+\frac{R_{s}}{L_{s}}i_{s}\left(t\right)+\frac{1}{L_{s}}\dot{\phi }\left(t\right)-\frac{1}{L_{s}}v_{s}\left(t\right)=0\\ \ddot{\phi }\left(t\right)+\frac{1}{C_{g}R_{d}}\dot{\phi }\left(t\right)+\frac{a}{C_{g}}\phi \left(t\right)+\frac{b}{C_{g}}\phi^{p}\left(t\right)-\frac{1}{C_{g}}i_{s}\left(t\right)=0 \end{array}\right.,$$

where the non-linearity is due to the term $\frac{b}{C_{g}}\phi^{p}\left(t\right)$ with p>1 and the solution relies on the specific prefault operation conditions, i.e., ϕ(t=0), $\dot{\phi }\left(t=0\right)$, $i_{s}\left(t=0\right)$, and IVT primary voltage characteristics during the transient, e.g., the rise time and peak values of $v_{s}$.

Given Equation (3), the mathematical analysis of ferroresonance phenomena is complicated, but, for detection purposes, some of their effects are well known:

- High distortions of waveforms, overvoltages, and overcurrents.
- Neutral voltage displacement.
- Overheating of transformers.
- Noise and vibration in transformers.
- Damage to transformer primary winding and electrical equipment.

However, the above consequences can also be produced by other sort of things (e.g., faults or imbalances due to switching operations), and, as a consequence, obtaining a reliable ferroresonance detection algorithm is challenging.

**Figure 3:**
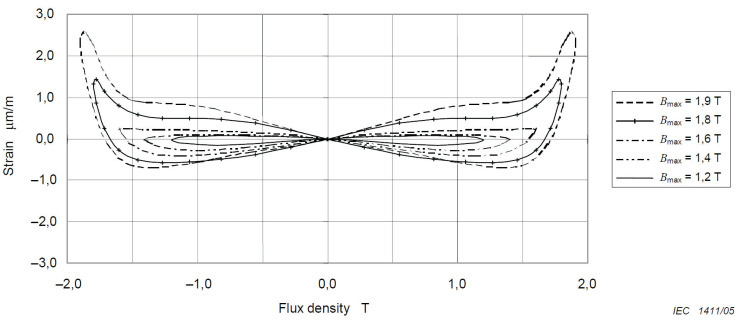
Example curves showing relative change in length for one type of core lamination during complete cycles of applied 50 Hz AC induction up to different peak flux densities ($B_{max}$ = 1.2T– 1.9T) [35].

During ferroresonance events, the IVT becomes saturated, and the magnetic field fluctuates significantly. Due to these fluctuations and the magnetostriction, the size of ferromagnetic elements changes. Consequently, vibrations and an audible noise, which are distinctively different from and louder than the normal operation noise, appear in the IVT. The characteristics of the resulting noise, both spectrum and intensity, will depend on the magnetic properties of the core material, the magnetic flux density, the materials used to encapsulate the IVT, the mounting method, and the switchbox acoustic characteristics. Figure 3 shows that the higher the magnetic flux density is, the greater the voltage and acoustic noise are. Thus, when ferroresonance occurs, the vibration and the acoustic noise of the IVT will increase [35].

### 2.2. Indirect Detection through IVT Vibrations

**Figure 4:**
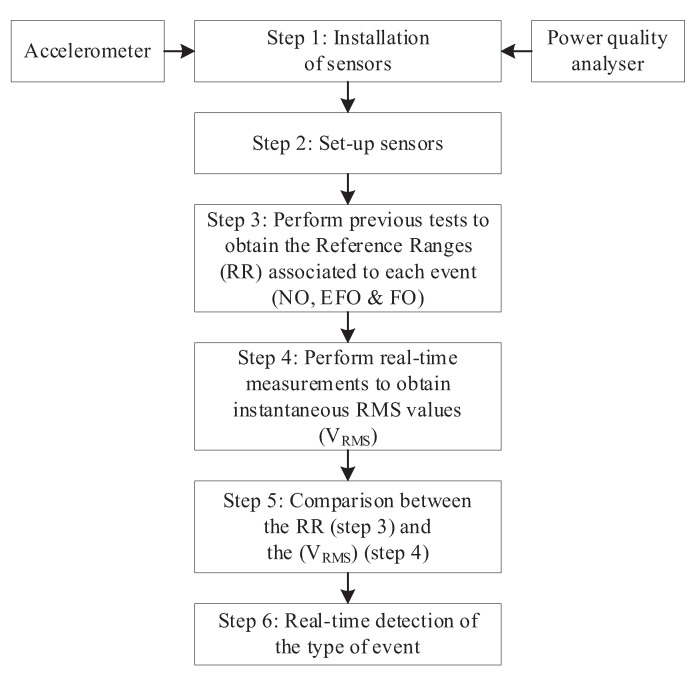
Methodology described in [32].

Among other indirect detection methods, the methodology proposed in [32] is based on measuring the IVT vibrations during the ferroresonance events by means of accelerometers. The obtained measurements are processed at different frequency bands, and the resulting RMS value is used, in comparison with predefined detection levels, to differentiate between normal (NO), fault (EFO), and ferroresonance operation (FO) conditions. The complete methodology is depicted in Figure 4, and the following steps are carried out:

- **Step 1**: Installing the accelerometers. Three IVTs are used for voltage measurement in isolated neutral power systems. One accelerometer must be located on the IVT case. Safety procedures and isolation levels for medium voltage grids must be accomplished. Special care must be taken to ensure an appropriate mechanical coupling.
- **Step 2**: Set-up sensors. Sensors must be calibrated to achieve their right operation at the IVT specific place. It will be necessary to calibrate the sensors to adjust the sensitivity, the range of measurements, etc. The auxiliary power supply must also be adjusted.
- **Step 3**: Perform previous tests to obtain the reference ranges (RR) associated with an IVT electrical event: normal operation (NO), single phase-to-earth fault operation (EFO), and ferroresonance operation (FO).
  - Step 3.1: Perform previous tests to measure the vibrations for each electrical event of the IVT.
  - Step 3.2: Perform the vibration signal conditioning for each electrical event of the IVT.
  - Step 3.3: Perform the discretization of the conditioned vibration signals for each electrical event of the IVT.
  - Step 3.4: Perform the equalization of the discretized vibration signals for each electrical event of the IVT.
  - Step 3.5: Obtain the RMS vibration signals from the equalized signals associated with each electrical event of the IVT.
  - Step 3.6: Obtain the RR associated with each electrical event of the IVT.
- **Step 4**: Perform real-time measurements to obtain instantaneous RMS values $V_{RMS}$.
- **Step 5**: Comparison between the RR (Step 3) and the instantaneous RMS values $V_{RMS}$ (Step 4).
- **Step 6**: Real-time detection of the type of event.

## 3. Indirect Ferroresonance Detection through Acoustic Noise

Assuming the methodology in [32], the accelerometers used as vibration sensors are replaced in this manuscript by acoustic noise ones, i.e., microphones. Firstly, the applicability of microphones for detection of ferroresonances is evaluated, and then, the methodology in [32] is modified to use microphones.

### 3.1. Acoustic Noise Sensors for IVT

**Figure 5:**
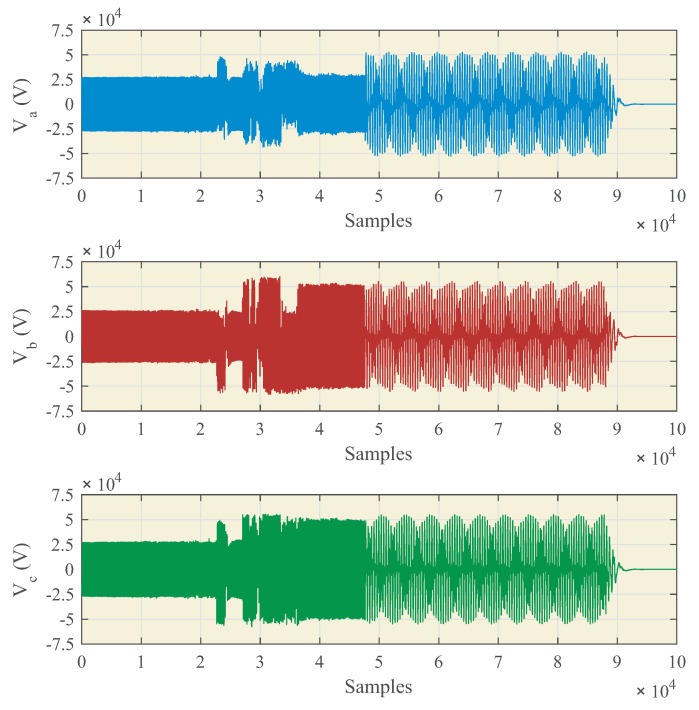
Line-to-earth fault and ferroresonance event. Time-domain phase voltages.

**Figure 6:**
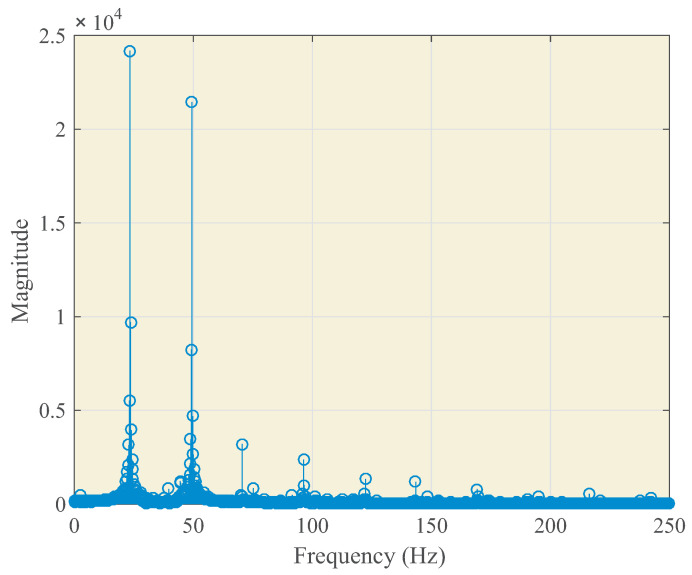
Line-to-earth fault and ferroresonance event. Spectrum of phase A voltage during the ferroresonance event.

Ferroresonance events stimulate the vibrations of the IVT, and given the manufacturing procedure and materials used, certain vibration modes are reinforced or attenuated, resulting in audible noise. The switchgear where the IVT is placed functions as a sound box, and the resulting noise depends on their mechanical characteristics. Then, the time and frequency domain characteristics of this noise depend on the characteristics of the ferroresonance event and the acoustic properties of both the IVT and the switchgear. In order to select the most suitable acoustic noise sensor, nine ferroresonance events in MV power systems, registered with power quality analyzers at different locations, were studied. The characteristics of the events depend, mainly, on the line-to-ground fault characteristics, the ratio generation/loading, and the line-to-ground capacitance. Figure 5 shows the measured feeder voltages at a sampling rate of 10 kHz for one of these events. After the fault is cleaned by the protections, the ferroresonance event occurs. Figure 6 is obtained by processing the spectra of phase voltages during the ferroresonance event. Most of the recorded events show subharmonic ferroresonances with the most significant frequency peak at 25 Hz, but harmonic ferroresonances were also recorded, with contributions up to 1 kHz, which matches the experimental results in the technical literature.

Given the aforementioned characteristics and the fact that the sensor must be placed in a switchgear in an MV substation or transformation center, the selected microphone must be small enough and the installation method must be simple and tolerant to errors, e.g., misalignment. Therefore, it must provide high sensitivity at a [20 Hz, 1 kHz] frequency range, it must be robust and stable enough, and it must avoid the effect of other electrical equipment. Moreover, since the detection strategy in [32] uses relative values due to different frequency bands, low-cost microphones can be potentially used despite of their poor characteristics.

From the frequency and sensitivity requirements, infra-sound microphones, e.g., Brüel & Kjær Type 4964 microphones [36], are the most suitable ones, but the prices can be relatively high, i.e., hundreds of USD. Both low-cost dynamic and condenser microphones will exhibit issues around 20 Hz, despite the available solutions to extend the frequency range below this value, e.g., [37,38]. Neglecting the frequency response, the robustness of dynamic ones, both the moving coil and the ribbon, the absence of polarization or self-noise would make piezoelectric microphones [39] valuable candidates for this application. However, magnetic fields within the switchgear, vibrations (e.g., switchgears in industrial environments), the high sensitivity, and the low price of condenser ones make electret microphones the most suitable ones for massive deployment.

A CMA-4544PF-W electret condenser microphone with pre-amplifying stage built-in around the low-noise OA MAX4466 from Maxim Integrated [40] was evaluated for ferroresonance detection purposes by means of LTspice from analog devices. This sensor can be purchased for USD 6.95 at [41]. Since the CMA-4544PF-W includes a FET transimpedance conversion stage [42] and the frequency response is flat within the interval of interest, the capsule can be approximated with a voltage-controlled current source [43], where the FET modulates the output current depending on the sound wave pressure and the microphone current.

Output current changes due to sound wave pressure are parameterized by means of the typical microphone sensitivity, $S_{typ}$, and the typical output impedance, $R_{o}$:

(4) $$Y_{o}=\frac{10^{-\frac{S_{typ}}{20}}}{R_{o}}.$$

For simulation purposes, the sound wave pressure, in Pa, is modeled through a voltage source; i.e., 1 Pa is equivalent to 1 V.

**Figure 7:**
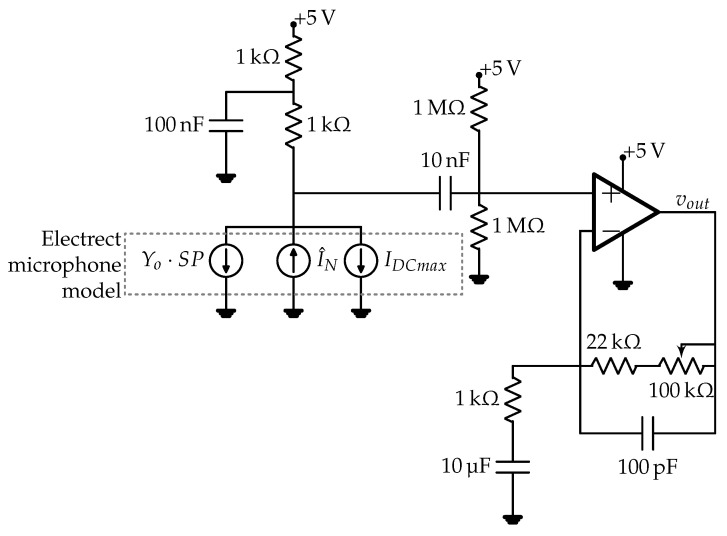
Circuit model of CMA-4544PF-W electret condenser microphone with pre-amplifying stage built in around the low-noise OA MAX4466.

The microphone self-noise is modeled through a constant current sink, which is parameterized through the signal-to-noise ratio, $SNR_{I}$ in the datasheet

(5) $$I_{N}=\frac{Y_{o}}{10^{\frac{SNR_{I}}{20}}},$$

which results in the current noise spectral density ${\hat{I}}_{N}^{\prime }=\frac{I_{N}}{\sqrt{BW_{A-w}}}$, where the bandwidth of the A-weighted noise distribution is approximated with 13.5 kHz. Since $SNR_{I}$ in the datasheet is obtained with a measurement resistor $R_{M}=$ 2.2 kΩ, its effect on ${\hat{I}}_{N}^{\prime }$ must be compensated for modeling purposes:

(6) $${\hat{I}}_{N}=\sqrt{{\left({\hat{I}}_{N}^{\prime }\right)}^{2}-\frac{4K_{B}T}{R_{M}}},$$

where $K_{B}$ is the Boltzmann’s constant, and *T* is the absolute temperature at which the $SNR_{I}$ is given in the datasheet. The obtained model parameters for this microphone are shown in Table 1, and the equivalent circuit model for this microphone and with pre-amplifying stage is depicted in Figure 7.

**Figure 8:**
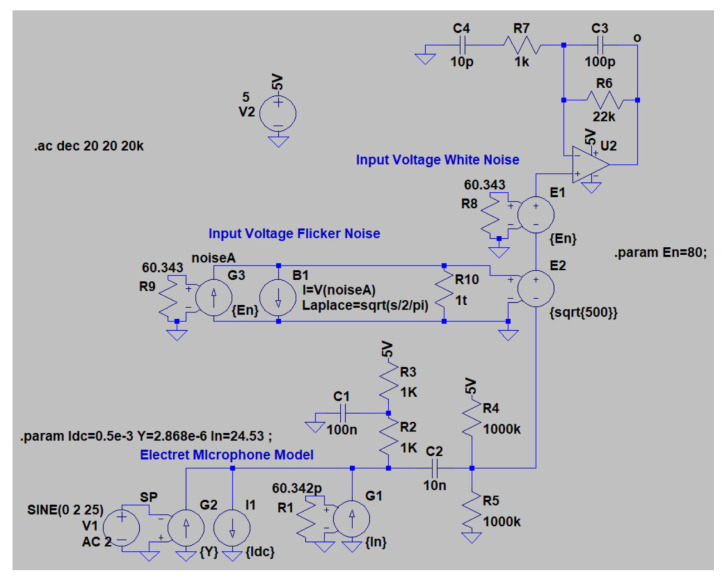
Simulation model in LTspice.

The frequency response and noise analysis were carried out in LTspice (Figure 8). The relevant datasheet parameters for OA MAX4466 are given in Table 2. These parameters are included in the simulation model by means of the symbol *universal op amp 2* in LTspice, and both input voltage and flicker noises are considered for noise analysis. Detailed info around noise considerations in operational amplifiers can be found in [44,45,46], which provides recommendations about integration of noise sources in LTspice simulation models.

**Figure 9:**
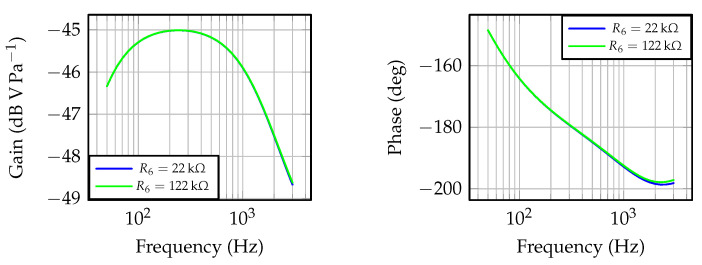
Frequency response of the CMA-4544PF-W electret condenser microphone with pre-amplifying stage built-in around the low-noise OA MAX4466.

The frequency responses for two extreme values of $R_{6}$ are depicted in Figure 9. The frequency range for simulation purposes matches the frequency range at which the microphone response is linear, i.e., 50 Hz and 3 kHz (from the microphone datasheet), ensuring the model validity assumptions in Figure 8 are met. Similar frequency responses are achieved within the microphone frequency range, with a −45.01 $\mathrm{dB}V\;{\mathrm{Pa}}^{-1}$ maximum gain at 251.8 Hz, for both $R_{6}=22\;k\Omega$ and $R_{6}=122\;k\Omega$. The responses differ slightly at frequencies above 1 kHz, and the minimal gain is −48.62 $\mathrm{dB}V\;{\mathrm{Pa}}^{-1}$, with $R_{6}=22\;k\Omega$. Considering sound pressure levels between barely audible (≈20 mPa) and pain (≈60 Pa), the evaluated microphone with a pre-amplifying stage would result in output voltages with peak-to-peak values across 0.14 mV and 674.04 mV, respectively.

The noise analysis is carried out by means of the *.noise* directive in LTspice, for the microphone frequency range resulting in a linear response, i.e., 50 Hz and 3 kHz. The output noise at the pre-amplifying stage output, at T=300 K, varies from 5.77 μV/Hz, with $R_{6}=22\;k\Omega$, to 6.18 μV/Hz ($R_{6}=122\;k\Omega$), which results in a maximum 0.05 mV peak-to-peak noise at the output. From the previous analysis around the gain and the sound pressure levels, the achieved output voltage noise levels are compatible with the signals to be measured. The noise analysis also reveals that the OA MAX4466 contributes the most to the output noise, and, if needed, it should be replaced with a quieter operational amplifier. It should also be considered that the power supply used must contribute to keeingp the output voltage noise below the required levels.

### 3.2. Modifications to the Methodology in [32] for Using Microphones as Sensors

Replacing the vibration sensor in [32] with the CMA-4544PF-W electret condenser microphone with the pre-amplifying stage requires the output voltage signal in Figure 7 to be conditioned properly. Since the analog-to-digital conversion (ADC) is carried out with a 12-bit 0–5 V successive approximation register (SAR) ADC, at a 10 kHz sampling rate, and given that the output frequency range of the microphone is [20 Hz, 20 kHz], a 4th-order low-pass Butterworth filtering stage, i.e., Equation (7), is used as an anti-aliasing filter, and the band-pass is amplified to maximize the resolution due to the signal discretization. Circuit-design considerations for driving the SAR-ADC can be found in [47].

(7) $$G_{LPF}\left(s\right)=\frac{6.234\;\cdot \;10^{15}}{s^{4}+1.64\;\cdot \;10^{4}s^{3}+1.35\;\cdot \;10^{8}s^{2}+6.48\;\cdot \;10^{11}s+1.56\;\cdot \;10^{15}}$$

**Figure 10:**
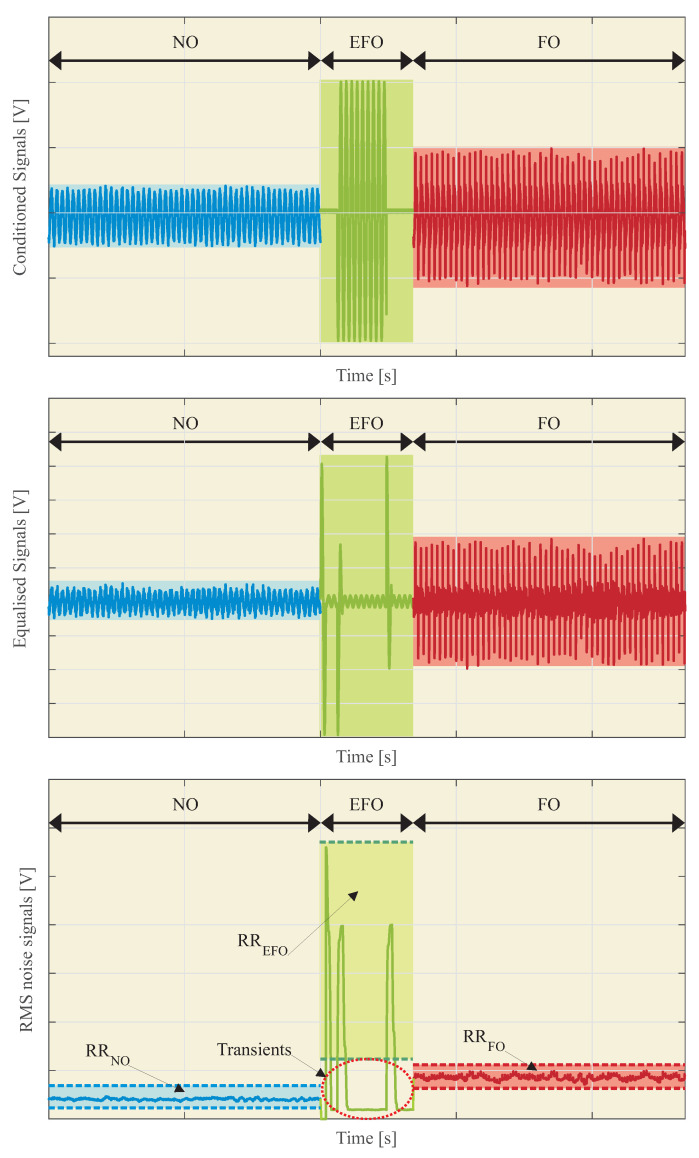
Example of results for conditioned, equalized, and RMS noise signals during NO, EFO, and FO of the IVT.

Some of the methodology steps in Section 2.2 must be modified to be used with microphones:

- **Step 1**: The microphone must be placed inside the switchgear, ensuring a proper fixation to the cabinet door.
- **Step 3.6**: The reference ranges, RR, for detection purposes, must be adjusted to the new sensor. Previous analysis allows the ranges to be approximated, but experimental tests are recommendable for fine tuning. Figure 10 shows an example. The RR associated with each electrical event *i*, i.e., $RR_{i}$, is calculated as the minimum and maximum of the RMS noise signal values. Thus,
  (8) $$RR_{i}=\left[RR_{i}^{min}-RR_{i}^{max}\right]$$
  where *i* is the electrical event associated with the RMS sound signal: NO, EFO, or FO.
  The transients during the EFO should be avoided. Therefore,
  (9) $$RR_{EFO}^{min}\approx RR_{FO}^{max}$$

## 4. Set-Up Facility

**Figure 11:**
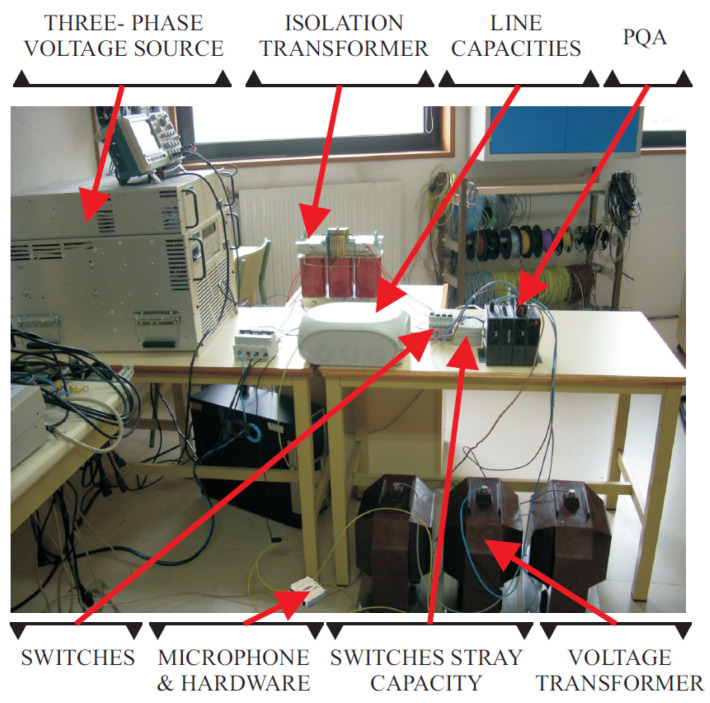
System description.

**Figure 12:**
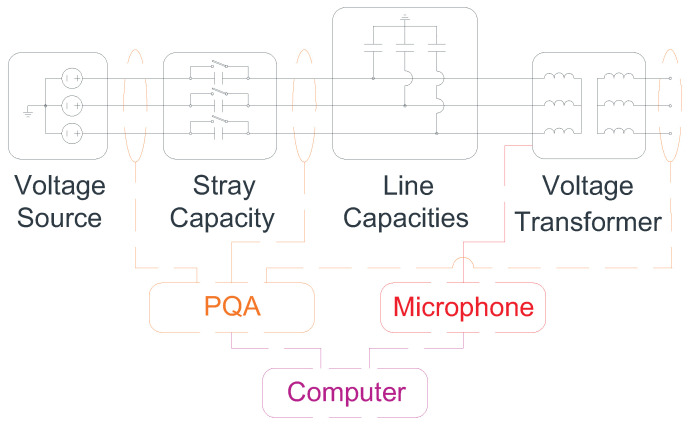
System block diagram.

The selection of the devices used to create the set-up facility is based on the two main requirements to achieve the aim of the paper: the creation of the ferroresonance and the data collection. As explained in the Introduction, for the ferroresonance occurrence, an isolated neural system (isolation transformer), capacitances (stray capacities of the switches and line capacitances), saturable inductances (inductive voltage transformer (IVT)), an energy source (voltage source), low losses, and the appearance of a transient (a fault) are required. For the data collection, a microphone to measure the sound of the IVT and a Power Quality Analyser (PQA) to measure the voltage and current signals of the system are installed. The elements used, the architecture of the set-up facility, and a block diagram are shown in Table 3, Figure 11 and Figure 12, respectively.

## 5. Experimental Results

### 5.1. Indirect Detection Method with Acoustic Noise Sensors

The methodology in Section 3.2 is used, step-by-step, to detect ferroresonance occurrence based on the IVT acoustic noise:

**Step 1**: Installation of sensors. The microphone was placed near the IVT (100 mm). The PQA was placed in different positions to compare results and to detect the ferroresonance.

**Step 2**: Set-up of sensors. All sensors were calibrated for an accurate measurement. In the signal conditioning stage, the gain is adjusted, and in the equalization stage the digital filters are adjusted.

**Figure 13:**
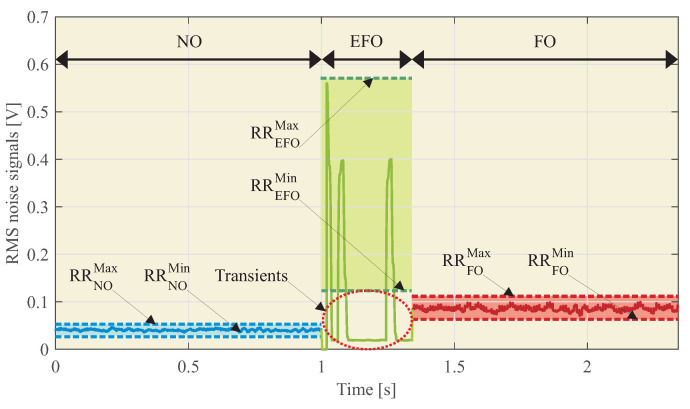
RMS sound values for NO, EFO, and FO of the IVT.

**Step 3**: Perform previous tests to obtain the RR associated with each electrical event of the IVT (Figure 13).

**Figure 14:**
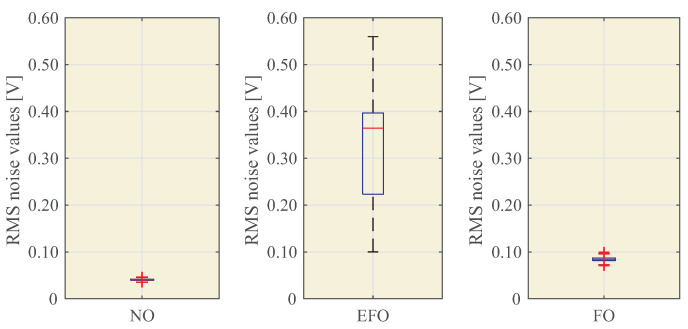
Box plots of the RMS sound values for NO, EFO, and FO of the IVT.

Figure 14 and Table 4 show the statistical data and the values: $RR_{NO}^{Min}$, $RR_{NO}^{Max}$, $RR_{EFO}^{Min}$, $RR_{EFO}^{Max}$, $RR_{FO}^{Min}$, and $RR_{FO}^{Max}$.

**Step 4**: Perform real-time measurements to obtain instantaneous RMS values $V_{RMS}$.

**Figure 15:**
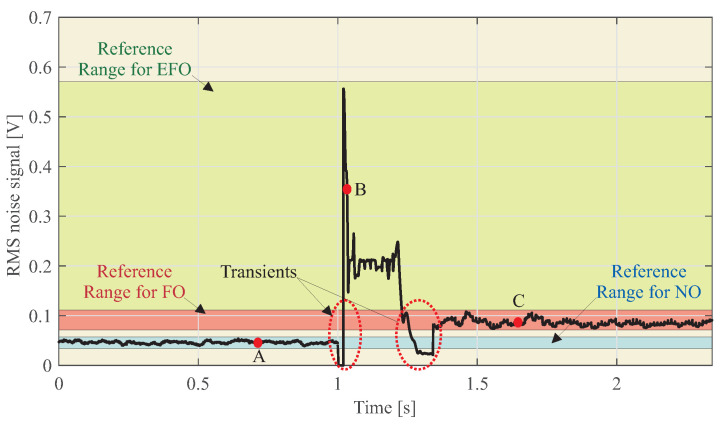
Real-time RMS sound signal.

Figure 15 shows a real-time RMS sound signal. In this figure, the RR associated with each type of event can also be seen.

**Step 5**: Comparison between RR and the real-time sound signal.

Taking into account the RR obtained in Table 4 and the real-time sound signal of Figure 15, it can be concluded that:

- From 0 to 1 s, the noise signal is in the NO event type.
- From 1 to 1.3 s, the noise signal is in the EFO event type.
- From 1.3 to 2.5 s, the noise signal is in the FO event type.

Figure 15 shows two zones of transients that are associated with variation owing to the line fault. These transients can be neglected because of their short duration.

**Step 6**: Real-time detection of the type of event.

This methodology can be used continuously over time to detect ferroresonance in real time.

### 5.2. Comparison of Indirect Methods with Accelerometers or Microphone

Both the vibration sensor in [32] and the proposed acoustic noise sensor, with the required signal conditioning circuitry, were compared experimentally using the setup in Figure 11. Line-to-ground faults with *C_g_* = 10 μF were tested with six different combinations of locations. Ensuring the synchronization of the measured signals is mandatory for comparison purposes. For this purpose, a National Instruments USB-6008 control card is used as a data logger, which, monitoring three signals, results in an effective sampling rate of 3.3 kHz per channel. The signals logged are the output voltages of the signal conditioning circuits for the acoustic sensor, $v_{o,an}$, and the vibration sensor, $v_{o,v}$, plus the measured IVT primary voltage through a 100 MHz 1500 V differential probe. Both the accelerometer and the microphone were placed at different locations and positions in order to evaluate the sensitivity to the installation procedure.

In tests #1–#3, the microphone was located inside the switchgear, and the accelerometer was placed on the faulty IVT, i.e., #1; the nearest IVT, i.e., #2; and the far one, i.e., #3. In tests #4–#6, the switchgear panel vibrating the most was identified and the accelerometer was coupled to it. The microphone was placed inside the switchgear in test #4, and outside, several centimeters away, in test #5 and at 0.3 m in test #6. Table 5 shows these placement combinations. The six ferroresonance events were similar, but not equal, since, from Equation (3), the ferroresonance voltage strongly depends on the initial conditions.

**Figure 16:**
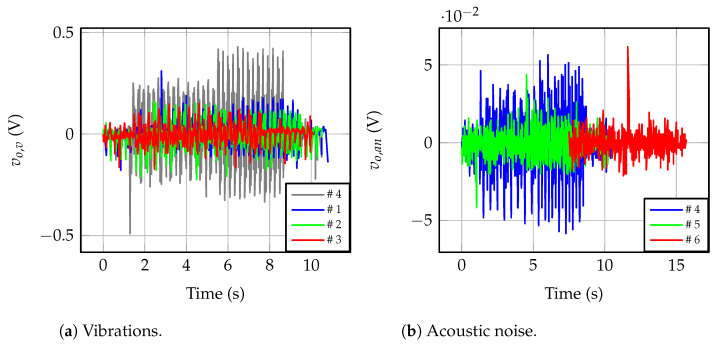
Output voltages for sensor locations in Table 5.

Figure 16a shows the measured voltage signals for the ferroresonance events tested. Waveforms are shifted across the time axis since the event characteristics are different in each test. Placing the accelerometer on the best inner panel of the switchgear, the greatest amplitude was measured, reaching 420 mV. If the accelerometer was directly placed on the IVT case, the vibrations are attenuated. If the measured IVT is the same where the fault occurs, i.e., #1, the 310 mV peak is measured when the fault occurs. The worst measurement is achieved if the accelerometer is placed on the far IVT, with only 310 mV peak. Figure 16b shows the results obtained with the microphone. In comparison, the magnitude is two orders of magnitude smaller, since only the pre-amplifying stage is used in these tests. As can be seen, byplacing the microphone inside the switchgear, the best results are obtained, with a 532 mV peak. The worst results are due to placing the microphone outside, at 0.3 m, resulting in 195 mV peak during the ferroresonance (a 612 mV peak occurs when the ferroresonance starts). Considering both figures, the amplitude variation of the measurement due to the worst and the best sensor locations are 26.2% and 63.3% for the accelerometer and the microphone, respectively.

**Figure 17:**
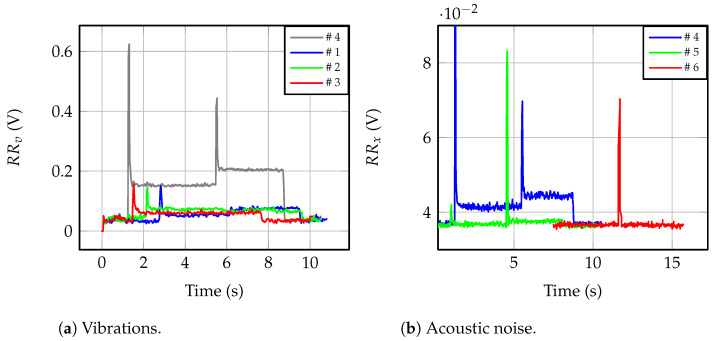
Effective values for detection purposes (RR) for locations in Table 5.

Further analysis is given in Figure 17, where the RMS values of the signals used for detection purposes are depicted. Figure 17a shows that by placing the accelerometer on the best inner panel of the switchgear, i.e., test #4, NO, EFO, and FO states can be resolved. The measured average values are 39.6 mV, 152.6 mV, and 204.8 mV for NO, EFO, and FO in test #4, respectively. The worst resolution due to the accelerometer occurs if placed on the far IVT case, and then, the measured average values are 37.8 mV, 60.2 mV and 60.7 mV for NO, EFO, and FO, respectively, in test #3. From the measured values for EFO and FO in test #3, both operation modes result in similar mechanical vibrations on the far IVT. RMS values are also plotted for the microphone, in Figure 17b. By placing the microphone on the inner door panel of the switchgear, the greatest resolution is achieved, 36.8 mV, 40.3 mV, and 44.5 mV for NO, EFO, and FO, respectively, in tests #1 to #4. In test #6, the microphone is located outside the switchgear, at 0.3 m, and the measured RMS values are 36.3 mV, 36.6 mV and 36.6 mV for NO, EFO, and FO, respectively. Differences among all three operation modes are negligible in test #6.

## 6. Discussion

A fast and accurate detection of the occurrence of ferroresonances in MV power systems allows the distribution system operators to isolate and mitigate the phenomenon. Detection methods based on electrical variables are accurate but relatively expensive for massive deployment of sensors and relays in transformation centers; indirect detection methods can be used as a cost-effective solution. This manuscript evaluates the acoustic noise sensors to reveal the occurrence of ferroresonances. Table 6 compares different ferroresonance-detection methods. Compared to vibration measurements using accelerometers, sensors based on electret microphones are cheaper and allow for fast and accurate detection if placed inside the switchgear. In addition, the proposed sensor is less prone to inaccuracies due to the installation procedure. The proposed sensor costs less than USD 10.

As for the massive deployment of the proposal, power-quality issues complicate the identification of ferroresonance events. Extraordinarily high voltage-harmonic distortion may also result in audible noise levels within the frequency band of the proposed sensor, as shown in [48], where a 150 kVA distribution transformer for a data center, operating at 0.7 power factor and $THD_{I}=16.4\%$, results in increasing the audible noise by 13.6dB, mainly in the frequency range of [0.1, 1.5] kHz. Interhamonic interaction can also change the vibration and acoustic noise pattern of the IVT. Low-frequency interharmonics, e.g., below 20 Hz, affect this pattern more [49]. However, it should be noted that these power quality issues are more relevant to the identification methodology for revealing the ferroresonance than the sensor proposed.

The proposed technique is described, and its performance is analyzed using an experimental test bench. The results obtained show the feasibility of the proposed detection technique. As shown in Figure 13, the system detects different patterns for normal operation (NO), single phase to earth fault operation (EFO) and ferroresonance operation (FO). The box plots of Figure 14 show that the levels of RMS noise are very different from EFO to FO, allowing Reference Ranges (RR) to be created for each event and, in real-time detection, to distinguish between a single phase to earth fault and ferroresonance. Experimental results show that accelerometer-based measurements within the switchgear are more sensitive to the installation procedure than the microphone. Just placing the microphone outside the switchgear does not result in any identification. If accelerometers are used for ferroresonance detection, one accelerometer should be used per IVT in order to decouple the effect of the mechanical adjustment of the switchgear, which increases the cost of this solution.

## Figures and Tables

**Table 1:** Model parameters for CMA-4544PF-W electret condenser microphone.

Datasheet Param.	Value	Model Param.	Value
Sensitivity ($S_{typ}$)	−44 dB	Output admittance ($Y_{o}$)	2.868 μA/$V^{-1}$
Output impedance ($R_{o}$)	2.2 kΩ		
Signal-to-Noise Ratio ($SNR_{I}$)	60 dB	Current noise	24.53 pA/$\sqrt{\mathrm{Hz}}$
Current consumption ($I_{dc,max}$)	0.5 m A	spectral density (${\hat{I}}_{N}$)	

**Table 2:** Model parameters for MAX4466 from Maxim Integrated.

Datasheet Param.	Value
Open-Loop Gain ($A_{VOL}$)	125 dB ($R_{L}=$ 100 kΩ)
Gain Bandwidth (GBW)	125 kHz
Slew Rate (SR)	300 mV μs^−1^
Input Noise Voltage Density (${\hat{V}}_{N}$) (f= 1 kHz)	80 nV/$\sqrt{\mathrm{Hz}}$
Voltage noise 1/f corner frequency	500 Hz

**Table 3:** List of main elements of the system.

Element	Description and Technical Data
Microphone	To measure the sound of the IVT. The selected microphone was an omnidirectional electret condenser microphone from CUI Inc. with a −44 dB sensitivity, an operating frequency in the range (0.02, 20) kHz.
PQA	To analyze current and voltage.
Line capacities	To model the line (from 1 to 50 µF) capacities and to generate different ferroresonance types.
Voltage source	To feed the IVT with AC voltage (4 kVA).
IVT	220/$\sqrt{3}$:110/$\sqrt{3}$ V and 5 VA.
Switch stray capacity	To simulate the parasitic capacitance of grid switches.
Switches	To simulate the switches of the grid.
Isolation transformer	To make an isolated neutral system.

**Table 4:** List of reference ranges RR for NO, EFO, and FO of the IVT.

Value	NO	EFO	FO
$RR^{Min}$	0.035	0.100	0.070
$RR^{Max}$	0.047	0.559	0.099

**Table 5:** Comparison tests carried out.

	Accelerometer				Microphone		
Test No.	Faulty	Close	Far	Inner	Inside	Outer	at
	IVT	IVT	IVT	SG ${}^{1}$ Panel	SG	SG Panel	0.3 m
# 1	X				X		
# 2		X			X		
# 3			X		X		
# 4				X	X		
# 5				X		X	
# 6				X			X

^1^: switchgear.

**Table 6:** Advantages and disadvantages of existing ferroresonance-detection methods.

Measured Parameter	Advantages	Disadvantages
Voltage waveforms	High accuracy in ferroresonance detection.	High sample rate and precision requirements. Isolation problems. High cost.
Temperature		Isolation problems. Low accuraccy in ferroresonance detection.
Vibrations	Low cost. High accuracy in ferroresonance detection.	Isolation problems. Regular calibration. Accuracy sensitive to the installation procedures.
Acoustic Noise	Low cost. High accuracy in ferroresonance detection. Accuracy less sensitive to the installation procedures. No isolation problems.	Sensitive to the microphone location.

## Abbreviations

The following abbreviations are used in this manuscript:

$A_{VOL}$	Operational amplifier open-loop gain [dB].
ADC	Analog-to-digital converter.
$BW_{A-w}$	Bandwidth of the A-weighted noise distribution [Hz].
EFO	Earth fault operation.
ϕ	IVT magnetic flux [T].
FET	Field-effect transistor.
FO	Ferroresonance operation.
$G_{BW}$	Operational gain bandwidth [Hz].
$G_{LPF}\left(s\right)$	Low-pass filtering transfer function.
${\bar{I}}_{C_{g}}$	Capacitive current [A].
$I_{dc,max}$	Microphone current consumption [A].
$i_{L}$	IVT current [A].
${\bar{I}}_{L}$	Phasorial representation of IVT current [A].
${\hat{I}}_{N}^{\prime }$	Microphone uncompensated current noise spectral density [$A/\sqrt{\mathrm{Hz}}$].
${\hat{I}}_{N}$	Microphone compensated current noise spectral density [$A/\sqrt{\mathrm{Hz}}$].
$i_{s}$	Line current [A].
${\bar{I}}_{s}$	Phasorial representation of line current [A].
IVT	Inductive voltage transformer.
$K_{B}$	Boltzmann’s constant [$J/K^{-1}$].
LPF	Low-pass filter.
NO	Normal operation
OA	Operational amplifier.
PQA	Power quality analyser.
PS	Power system.
$R_{d}$	Damping resistor [Ω].
$R_{o}$	Microphone typical output impedance [Ω].
RMS	Root mean square.
RR	Reference range.
$RR_{i}$	Reference range for the operation *i*.
$RR_{i}^{min}$	Minimum value of the reference range for the operation *i*.
$RR_{i}^{max}$	Maximum value of the reference range for the operation *i*.
$S_{typ}$	Typical microphone sensitivity [dB].
SAR	Successive-approximation register.
SNR	Signal-to-noise ratio [dB].
SP	Sound wave pressure [Pa].
SR	Operational amplifier slew rate [$V\;s^{-1}$].
*T*	Temperature [K].
${\hat{V}}_{N}$	Operational amplifier input noise voltage density [$V/\sqrt{\mathrm{Hz}}$].
$v_{s}$	Line-to-ground voltage [V].
$V_{s}$	RMS value of line-to-ground voltage [V].
${\bar{V}}_{s}$	Phasorial representation of line-to-ground voltage [V].
${\bar{X}}_{C_{g}}$	Line-to-ground reactance [Ω].
${\bar{X}}_{L_{IVT}}$	IVT reactance [Ω].
${\bar{X}}_{s}=R_{s}+j\omega L_{s}$	Line impedance [Ω].
$Y_{o}$	Microphone output admittance [$A\;V^{-1}$].
